# GYY4137 Promotes Mice Feeding Behavior via Arcuate Nucleus Sulfur-Sulfhydrylation and AMPK Activation

**DOI:** 10.3389/fphar.2018.00966

**Published:** 2018-08-21

**Authors:** Jun Zhou, Xiao-Hui Lv, Jun-Juan Fan, Li-Yun Dang, Kun Dong, Bo Gao, Ao-Qi Song, Wen-Ning Wu

**Affiliations:** ^1^Department of Pharmacy, Xi’an Chest Hospital, Shaanxi University of Chinese Medicine, Xi’an, China; ^2^Department of Pharmacology, School of Basic Medical Sciences, Key Laboratory of Anti-inflammatory and Immunopharmacology, Anhui Medical University, Hefei, China

**Keywords:** hydrogen sulfide, arcuate nucleus, GYY4137, food intake, AMPK, sulfur-sulfhydrylation

## Abstract

Hydrogen sulfide (H_2_S) is an endogenous gaseous molecule and plays important biological and neurochemical roles in many processes such as the neural activity and immunity. The arcuate nucleus (ARC) of hypothalamus is a control center for appetite and energy metabolism. AMPK is a gage kinase in the monitoring of energy status and regulation of energy metabolism, and it can be activated by H_2_S via CaMKKβ/AMPK pathway. But the role of H_2_S in ARC and appetite has not been reported. Here we studied the orexigenic effect of H_2_S and the mechanisms by means of GYY4137, a water soluble and slow-releasing donor of H_2_S, and protein sulfur-sulfhydrylation analysis. We demonstrated that GYY4137-derived H_2_S increased food intake of mice, augmented the production of neuropeptide Y (NPY), and elevated the protein sulfur-sulfhydrylation level and the activation of AMPK and CaMKKβ in ARC. Blocking sulfur-sulfhydrylation with DTT eliminated GYY4137-induced activation of AMPK and CaMKKβ. DTT and preventing AMPK activation in ARC with Compound C and Ara-A could both attenuate the orexigenic effect of GYY4137. These findings suggest that H_2_S enhances appetite through protein sulfur-sulfhydrylation and the activation of AMPK and NPY function in ARC.

## Introduction

Hydrogen sulfide (H_2_S) is produced endogenously in mammals by metabolism of sulfur-containing amino acids under the catalysis of special enzymes (Predmore et al., 2012; Sheibani et al., 2017) and has been regarded as the third bio-active gas or gaseous signaling molecule by accumulated evidence (Gadalla and Snyder, 2010; Predmore et al., 2012; Yu et al., 2017). H_2_S plays important physiological and pathological roles in many biological processes and acts as a critically functional regulator in several brain regions, such as the hippocampus and cerebral cortex (Giuliani et al., 2013; Yakovlev et al., 2017; Zhu et al., 2017). The arcuate nucleus (ARC) of hypothalamus is an important control center for appetite and energy metabolism (Joly-Amado et al., 2014; Reis et al., 2015). The effect of H_2_S on ARC and feeding behavior has not been explored.

Adenosine 5′-monophosphate (AMP)-activated protein kinase (AMPK) is an important modulator and gage molecule in energy metabolism and food intake (Oh et al., 2016; Dong et al., 2017). By sensing the available level of energetic substrates, AMPK determines the activation or suppression of orexigenic neurons such as neuropeptide Y (NPY) neurons and controls the production of NPY in ARC, the appetite regulation center in hypothalamus, and thus regulates feeding behavior (Blankenship et al., 2016; Carneiro et al., 2016). Ca^2+^/calmodulin-dependent protein kinase β (CaMKKβ) is a vital upstream activator of AMPK (Anderson et al., 2008), and it has also been reported that AMPK-mediated feedback phosphorylation of CaMKKβ regulates the CaMKKβ/AMPK signaling cascade and may be physiologically important for intracellular maintenance of Ca^2+^-dependent AMPK activation by CaMKKβ (Nakanishi et al., 2017).

As an endogenous signaling molecule, H_2_S exerts pivotal functions in many aspects, including immunity, metabolism, cardiovascular, and neural activity (Gadalla and Snyder, 2010; Gong et al., 2010; Predmore et al., 2012; Weber et al., 2016). Studies have reported that H_2_S elevates intracellular calcium level through glutamate receptor (Lee et al., 2006; Zhang and Bian, 2014) and increases AMPK activity (Lee et al., 2012; Zhou et al., 2014). By promoting AMPK activation, H_2_S exerts various physiological functions such as anti-inflammatory and cytoprotective effects. It is reported that CaMKKβ-dependent activation of AMPK is critical to the effects of H_2_S on neuroinflammation suppression and insulin sensitivity enhancement (Zhou et al., 2014; Chen et al., 2017; Wang et al., 2017). However, there is no report about the role of H_2_S-induced activation of AMPK in feeding behavior.

Resembling another retrograde gaseous neuromodulator nitric oxide and its nitrosylation effect, H_2_S is a sulfur-sulfhydrylation molecule and exerts its biological function mainly through protein sulfur-sulfhydrylation (Vandiver et al., 2013; Ju et al., 2015; Saha et al., 2016; Zhang et al., 2017). Similar to protein sulfur-nitrosylation, sulfur-sulfhydrylation by H_2_S influences functions of many proteins, such as potassium ion channel, pyruvate carboxylase, guanylate cyclase, and NF-κB (Mustafa et al., 2011; Paul and Snyder, 2012; Vandiver et al., 2013; Ju et al., 2015). But the effects of sulfur-sulfhydrylation on AMPK activation and food intake are unclear.

In the present study, we reported the orexigenic effect of GYY4137, a water soluble and slow-releasing donor of H_2_S, on mice and the underlying mechanism. We found that this effect of H_2_S is mediated by the sulfur-sulfhydrylation of ARC proteins, including AMPK and CaMKKβ, which subsequently increases the activation of AMPK and NPY function in ARC and food intake of mice.

## Materials and Methods

### Animals

Male C57BL/6J mice (aged 8–10 weeks) were used for the experiments in this study. Mice were singly housed in standard cages with a small PVC pipe for environmental enrichment. The colony room was maintained at constant temperature (22 ± 1°C) and humidity (60–70%) on a controlled 12-h light/dark cycle (lights on at 20:00). All experimental manipulations and tests were carried out during the dark cycle. Laboratory standard food for mice and water were available *ad libitum* in home cages. All experiments were conducted in accordance with the Guide for the Care and Use of Laboratory Animals as adopted and promulgated by the U.S. National Institutes of Health. The adult and neonatal mice were obtained from Laboratory Animal Center of Anhui Medical University. The use of animals for all experimental procedures was approved by the Animal Welfare Committee of Anhui Medical University.

### Chemicals and Agents

GYY4137 [morpholin-4-ium 4-methoxyphenyl(morpholino) phosphinodithioate], DTT (DL-dithiothreitol), AMPK inhibitors Compound C (6-[4-[2-(1-piperidinyl)eth-oxy]phenyl]-3-(4-pyridinyl)-pyrazolo[1,5-a]pyrimidine), Ara-A (ATP-mimetic, 9-β-D-arabinofuranoside), and MMTS (S-methyl methane thiosulfonate) were purchased from Sigma-Aldrich (St. Louis, MO, United States). Biotin-HPDP and HRP (horseradish peroxidase)-conjugated streptavidin were purchased from Thermo Scientific (Rockford, IL, United States). Primary antibodies for phospho-AMPKα (Thr172), AMPKα, phospho-CaMKKβ (Ser458/495), and CaMKKβ were bought from Cell Signaling Technology (Boston, MA, United States). Primary antibodies for NeuN (neuron-specific nuclear protein), c-Fos, and NPY were obtained from Santa Cruz Biotechnology (Santa Cruz, CA, United States). Primary antibody for GAPDH (glyceraldehyde-3-phosphate dehydrogenase), HRP-conjugated secondary antibodies, fluorescent secondary antibodies, and bovine serum albumin (BSA) were obtained from Beyotime Biotechnology (Shanghai, China). Other agents were all purchased from commercial suppliers. Biotin-HPDP was freshly dissolved in dimethysulfoxide (DMSO) and other drugs were prepared freshly with double-distilled water or buffer solutions to the final concentrations before application.

### Feeding Experiment

We investigated the effect of GYY4137, a water soluble and slow-releasing donor of H_2_S (Szabo and Papapetropoulos, 2017) on feeding behavior of mice. The mice were divided into five dosage groups and their food intake was detected (measured by weighing the consumption of food for mice) at 2 h after the intraperitoneal (i.p.) injection of GYY4137 (10% in saline, w/v). Then, under the treatment of GYY4137 at a final concentration of 150.4 mg/kg, the cumulative food consumptions of mice were measured at 1, 2, 4, and 8 h after drug administration. To explore the blocking effect of DTT, Compound C, and Ara-A on the orexigenic effect of GYY4137, mice were treated with the blocking drugs through intra-lateral ventricle (i.c.v.) injection 10 min before the administration of GYY4137. The mice in the control groups were all administrated with 0.9% saline, vehicle of the drugs. Food consumptions of mice treated with GYY4137 (100 nmol per mouse) via i.c.v. injection was also measured at 2 h after the administration. As for the detection and comparison of the S-sulfhydrylation levels in the ARC of the normal feeding and food restricted mice, the mice in food restricted group were fasted for 12 h, and then the ARC tissues were isolated for S-sulfhydrylation assay.

### Surgical Procedure and Microinjection

The cannula was implanted according to stereotaxic mouse brain atlas (Paxinos and Franklin, 2012) with some modifications. Mice were anesthetized with chloral hydrate (350 mg/kg, i.p.) and placed in a stereotaxic apparatus (Stoelting) with the bregma and posterior on the same level. The body temperature was maintained at 37.0°C by an electric incandescent lamp. A small hole was drilled into the bone to insert a cannula into the lateral ventricle (LV, 0.7 mm posterior to the bregma, 1.0 mm lateral to the midline, 2.2 mm vertical from the cranial theca). A stainless steel cannula (length 6 mm, OD 0.5 mm) was implanted into the place according to the coordinates located above. The cannula was fixed to the skull with the aid of dental acrylic resin and dental cement. Cannula placement was confirmed by analyzing the brain slices of mice cannula-implanted under magnifying glass after perfusion, fixation, and slicing (thickness 50 μm) of the brain. The experiments began after a 5-day recovery period from surgery. The drugs were injected into the lateral ventricle with a microsyringe (2 μL) connected by a PE-10 polyethylene tubing (10 cm) to a needle (OD 0.2 mm), which was introduced into the lateral ventricle through the cannula fixed to the head of mouse. The injection volume was set to 2 μL within a period of 2 min.

### Preparation of ARC Neurons

Primary cultures of hypothalamic ARC neurons were prepared as previously described with some modifications (Chen et al., 2012; Wu et al., 2013; Palomba et al., 2015). Briefly, C57BL/6J mice, 1–2 days postnatal, were humanely killed by decapitation. The ARC tissue was quickly dissected and transferred to PBS-buffered solution containing 135 mM NaCl, 5 mM KCl, 1 mM CaCl2, 1 mM MgCl2, pH 7.3, and finely chopped. Then the ARC tissues were treated with 0.125% trypsin in PBS-balanced solution for 20 min at 37°C and gently triturated using flame-polished Pasteur pipettes. Cell suspension was centrifuged for 7 min at 1000 *g*, and then the cell pellets were resuspended in the Dulbecco’s modified Eagle’s medium (DMEM) and F-12 supplement (1:1) with 10% fetal bovine serum before plating onto glass-bottomed dishes (MatTek) coated with poly-L-lysine (20 μg/mL for 1–2 h) and kept at 37°C in 5% CO_2_ incubator. After overnight incubation in DMEM, the medium was changed to neurobasal medium (Gibco) containing 15 mmol/L glucose supplemented with 2% B27, 2 mM glutamine, 10 μg/mL penicillin, and 10 μg/mL streptomycin. The ARC neurons were fed with fresh medium every 48 h. Microscopically, glial cells were not apparent in cultured cells employing this protocol. The neurons were maintained for 6–8 days in primary culture until used for subsequent cell experiments.

### Immunofluorescence Assay

After treatment, the mouse was anesthetized with chloral hydrate and continuously perfused through the left ventricle with 4% polyformaldehyde for 0.5–1 h. Then the whole brain was isolated and immersed into 4% polyformaldehyde for 24 h. The brain was sliced using oscillating slicer (Leica) and the brain slices were rinsed with 10 mM PBS. Then the slices were treated by permeabilization with 10 mM PBS containing 0.3% Triton X-100 (v/v) for 30 min, and then blocked with 3% BSA-PBS (w/v) for 1 h, and incubated with 1:50 anti-c-Fos and 1:50 anti-NeuN antibodies in 10 mM PBS containing 0.3% Triton X-100 and 1% BSA and 2% goat serum (v/v) overnight at 4°C. After washes with PBS, slices were incubated with 1:100 Rhodamine-conjugated goat anti-rabbit and FITC-conjugated goat anti-rat secondary antibodies in 10 mM PBS containing 0.3% Triton X-100 and 1% BSA and 2% goat serum for 1 h at room temperature. Finally, the slices were mounted on glass slides with 30% glycerin, and visualized and captured with a fluorescent microscopy (Olympus BX43, Japan) for the expression and co-localization of c-Fos and neuronal marker NeuN. The fluorescence quantification software is Scion Image 4.0 (Scion Inc., Fredrick, MD, United States). We took the c-Fos/NeuN ratio of their fluorescence intensity of each single cell as data to make comparison between the vehicle and GYY-treated groups.

### Measurement of NPY Content

The procedure for NPY content detection was executed as our previous study (Wu et al., 2013) with minor modification. After treatment for 1 h, mice were sacrificed by decapitation under 10% chloral hydrate anesthesia, and the entire ARC tissue was isolated from the brain. To minimize the deviation of operation-induced stimulation to the mice, we gave the control group with equal volume of saline injections and get the sample preparations for detection both at 1 h after treatment. Isolated tissues were washed twice with ice-cold PBS and then lyzed on ice in extraction buffer containing 50 mM Tris base (pH 7.4), 100 mM NaCl, 1% NP-40 (v/v), 10 mM EDTA, 20 mM NaF, 1 mM phenylmethylsulfonyl fluoride, 3 mM Na_3_VO_4_, and protease inhibitors. The homogenates were centrifuged at 12,000 *g* for 15 min at 4°C. Supernatant was separated and stored at -80°C until use. Protein concentration was determined using the BCA protein assay kit (Pierce Biotechnology, Inc.), and NPY content was measured by a commercial enzyme-linked immunosorbent assay kit (R&D Systems, Minneapolis, MN, United States) according to the manufacturer’s protocol. NPY content was expressed as picogramas per microgram of protein.

### Western Blot Assay

Western blot assay was processed as our previous studies with minor modifications (Li et al., 2016; Hou et al., 2017). The sample preparations for western blotting were lyzed solutions of the whole cells or tissue which include nuclear and cytosolic fractions. ARC tissues isolated from the brain of mice after treatment were homogenized rapidly in ice-cold lysis buffer containing protease and phosphatase inhibitors (as above) and centrifuged for 15 min (12,000 *g*, 4°C). The supernatant fraction was collected and stored at -80°C for western blot assay. Protein concentration was examined by BCA assay and 30 μg of protein sample was loaded in each lane for comparison, with GAPDH as loading control. Following SDS-PAGE electrophoresis, the proteins were transferred to polyvinylidenedifluoride membrane. After blocking with 5% BSA in Tris-buffered saline containing 0.1% Tween-20 (v/v) for 1 h at room temperature, transferred membranes were incubated overnight at 4°C with appropriately diluted primary antibodies against each protein below: anti-c-Fos (1:1000), anti-NPY (1:800), anti-phospho-AMPKα (Thr172; 1:500), anti-AMPKα (1:1000), anti-phospho-CaMKKβ (Ser458/495; 1:500), anti-CaMKKβ (1:1000), and anti-GAPDH (1:4000). Membranes were then incubated with HRP-conjugated secondary antibodies (anti-rabbit 1:2000, anti-rat 1:3000) for 1 h at room temperature before signals were visualized using the SuperSignal West Pico ECL kit (Thermo Scientific, Rockford, IL, United States). Images were captured with Micro Chemi (DNR Bio-imaging systems, Israel) and the optical density of the bands was determined using Scion Image software (Fredrick, MD, United States). All assays were performed at least three times. Results are presented as percentage of control after normalization (% control) in each blot to correct the variations between blots.

### Sulfhydrylation Detection

For the measurement of the sulfhydrylation levels of protein samples, the isolated tissue was homogenated in ice-cold lysis solution without EDTA. After centrifuged for 15 min (12,000 *g*, 4°C), the supernatant fraction was isolated and aliquoted into the 96-well plate. Equal volume of DTNB [5,5′-Dithio bis-(2-nitrobenzoic acid)] reaction buffer (10 mM MgCl_2_, 30 mM KCl, 25 mM Tris-HCl, 4 mM DTNB, pH 8.0) was also added to the wells and the reaction proceeded at 37°C in dark place for 15 min with moderate shaking. Then the absorbance of the wells was determined by a microplate reader at 415 and 595 nm. After deducting the blank control value, the absorbance value is directly proportional with the sulfydrylation level of the detected samples in a linear range.

### Sulfur-Sulfhydrylation Measurement

Protein sulfur-sulfhydrylation measurement was carried out according to previous studies (Paul and Snyder, 2012; Li et al., 2016; Saha et al., 2016) with some modifications. After treatment and dissection, the ARC tissues were collected and homogenated in special lysis solution prepared with HEN buffer (250 mM HEPES, 1 mM EDTA, 0.1 mM neocuproine; the pH was regulated to 7.7 with NaOH). The tissue homogenate was centrifuged (12,000 *g*, 4°C, 15 min) and 400 μL of supernatant was transferred into a boiling tube. Then, 1600 μL of selective sulfhydryl sealing agent (MMTS) solution [20 mM MMTS in HENS buffer (HEN buffer:25% sodium dodecyl sulfonatein H_2_O = 9:1)] was added into the boiling tube to selectively seal the free sulfhydryl of the total proteins. After 50°C water bath with slight shaking for 20 min, 100 μL of biotin-HPDP diluent (4 mM biotin-HPDP in DMSO) was then added into the boiling tube under darkness. Then the biotinylation reaction proceeded at 37 °C with slight shaking in dark place for 3 h. After that, the boiling tube was filled with -20°C prefrozen acetone, and then kept stewing in -20°C refrigerator for 30 min. Then the boiling tube was immediately centrifuged for 20 min (12,000 *g*, 4°C), and the acetone was poured and volatilized out. Finally, 100 μL of HEN buffer was used to redissolve the precipitation. Then the sulfur-sulfhydryl biotinylated protein sample was quantitatively determined, and then inactivated with non-reducing protein loading buffer. After SDS-PAGE electrophoresis, the proteins were transferred to polyvinylidenedifluoride membrane and blocked with 5% BSA for 1 h at room temperature. At last, the transferred membrane was incubated with HRP-conjugated streptavidin for 2 h at room temperature before signals were visualized using the ECL kit. As for the sulfur-sulfhydrylation detection of AMPKα and CaMKKβ, only a fraction of the total lysates was determined and loaded as references, and most of the protein samples were selectively biotinylated as above and precipitated by streptavidin-agarose beads for sulfur-sulfhydrylation proteins. Then the biotinylated proteins were eluted and detected by primary antibodies for western blot assay.

### Statistical Analysis

All data analysis was performed using SPSS 18.0 software (SPSS Inc., United States) and values were expressed as the mean ± SEM. Repeated measures analysis of variance (ANOVA) was used for the comparison of food intake between control and GYY groups at different time levels. One-way ANOVA and LSD *post hoc* test were used for comparisons between two or more groups. All tests for comparisons were set at α = 0.05 by two-side, and differences at the *P* < 0.05 level were considered statistically significant.

## Results

### GYY4137 Has Orexigenic Effect on Mice With Dosage and Time Dependence

**FIGURE 1:**
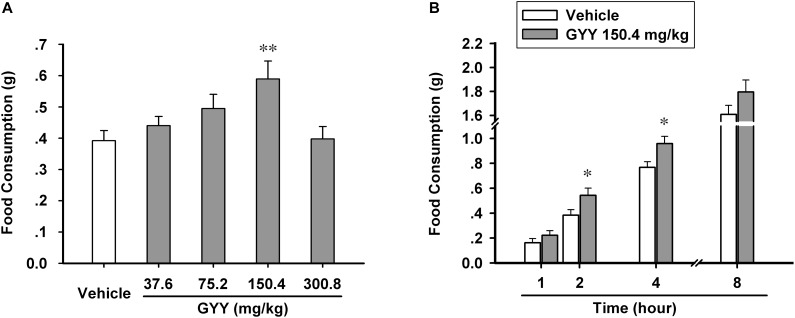
GYY4137 increases food consumption of male C57BL/6J mice. **(A)** GYY4137 influenced food intake of mice in 2 h after administration via i.p. at different physiological concentrations (*n* = 14). **(B)** GYY4137 at 150.4 mg/kg (400 μM) promoted food consumption of mice for 4 h after its administration (*n* = 8). Data are expressed as means ± SEM. One-way ANOVA and *post hoc* tests and repeated measures ANOVA were used. ^∗^*P* < 0.05, ^∗∗^*P* < 0.01 vs vehicle.

In order to explore the effect of GYY4137-dervied H_2_S on feeding behavior of mice and find the appropriate administration dosage, we divided the experimental animals homogenously into five groups (vehicle, 37.6, 75.2, 150.4, and 300.8 mg/kg) and tested their food consumption in 2 h after the application of GYY4137 or vehicle (saline) via i.p. injection. We found that GYY4137 augmented food intake in most of the dosage groups (37.6, 75.2, and 150.4 mg/kg), but with 150.4 mg/kg being the effective dosage [*n* = 14 mice for each group; *F*_(4,65)_ = 3.873, *P* = 0.009; vehicle, 0.392 ± 0.032; 37.6 mg/kg, 0.441 ± 0.030, *P* = 0.424 vs vehicle; 75.2 mg/kg, 0.495 ± 0.045, *P* = 0.081 vs vehicle; 150.4 mg/kg, 0.589 ± 0.057, *P* = 0.003 vs vehicle; 300.8 mg/kg, 0.398 ± 0.039, *P* = 0.926 vs vehicle; **Figure 1A**]. Then we asked how long time the orexigenic effect of GYY4137 could maintain. We measured the cumulative food consumption after GYY4137 (150.4 mg/kg) administration by four time point (1, 2, 4, and 8 h after application), and the result suggested that GYY4137 promotes feeding behavior of mice for hours and it significantly increased food intake in 2 and 4 h after the application [*n* = 8 mice for each group; 1 h, vehicle, 0.162 ± 0.032, GYY, 0.222 ± 0.037, *F*_(1,14)_ = 1.819, *P* = 0.219; 2 h, vehicle, 0.384 ± 0.044, GYY, 0.542 ± 0.058, *F*_(1,14)_ = 5.828, *P* = 0.046; 4 h, vehicle, 0.767 ± 0.045, GYY, 0.958 ± 0.058, *F*_(1,14)_ = 6.863, *P* = 0.034; 8 h, vehicle, 1.607 ± 0.077, GYY, 1.795 ± 0.100, *F*_(1,14)_ = 3.245, *P* = 0.115; **Figure 1B**].

### GYY4137 Increases the Activation of ARC Neurons and the Production of NPY

**FIGURE 2:**
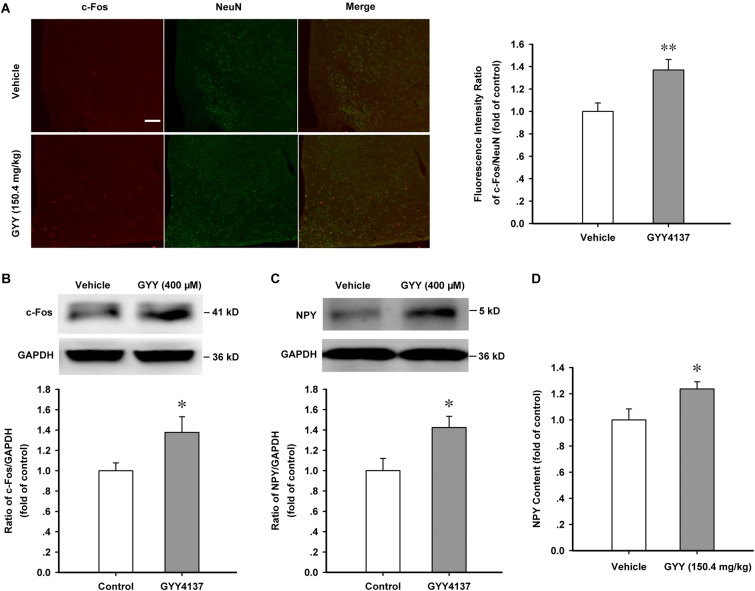
GYY4137 promotes the activation of ARC neurons and increases NPY content. **(A)** Immunofluorescence result showing GYY4137 (150.4 mg/kg) treatment for 1 h increased the nuclear expression of c-Fos in hypothalamic ARC neurons (vehicle, *n* = 18; GYY4137, *n* = 29 cells). Scale bar, 200 μm. **(B)** Western blot assay showing the increased expression of c-Fos protein after GYY4137 (150.4 mg/kg) treatment for 1 h in ARC tissue (*n* = 5). **(C)** Western blot assay showing GYY4137 (150.4 mg/kg, 1 h) increased the expression of NPY in hypothalamic ARC tissue (*n* = 4). **(D)** Statistical results showing GYY4137 (150.4 mg/kg, 1 h) increased the NPY content in ARC tissue of mice (*n* = 6). Data are normalized as folds to control and expressed as means ± SEM. One-way ANOVA was used. ^∗^*P* < 0.05, ^∗∗^*P* < 0.01 vs vehicle.

Arcuate nucleus is the regulation center of appetite and food intake and our previous study found that the percentage of NPY-positive neurons was about 57 ± 6.4 % in ARC neurons (Wu et al., 2013), thus we wanted to know if ARC neurons and NPY function are activated in the orexigenic effect induced by GYY4137. Immunofluorescence assay showed that GYY4137 (150.4 mg/kg) treatment for 1 h increased the nuclear expression of c-Fos in hypothalamic ARC neurons [vehicle, *n* = 18, 1.000 ± 0.076; GYY, *n* = 29, 1.370 ± 0.094; *F*_(1,45)_ = 7.697, *P* = 0.008; **Figure 2A**]. Western blot assay also showed that GYY4137 (150.4 mg/kg) increased the expression of c-Fos protein and NPY in the ARC tissue of mice [c-Fos: *n* = 5 mice for each group; vehicle, 1.000 ± 0.077; GYY, 1.377 ± 0.155; *F*_(1,8)_ = 5.347, *P* = 0.046; **Figure 2B**; NPY: *n* = 4 mice for each group; vehicle, 1.000 ± 0.120; GYY, 1.424 ± 0.109; *F*_(1,6)_ = 6.809, *P* = 0.039; **Figure 2C**]. ELISA assay also showed that GYY4137 (150.4 mg/kg) augmented the NPY content in ARC tissue [*n* = 6 mice for each group; vehicle, 1.000 ± 0.084; GYY, 1.236 ± 0.055; *F*_(1,6)_ = 5.591, *P* = 0.040; **Figure 2D**]. These results suggest that GYY4137 (400 μM or 150.4 mg/kg) promotes the activation of ARC neurons and the production of the orexigenic neuropeptide NPY.

### GYY4137 Enhances the Phosphorylation Level of AMPK and CaMKKβ in ARC

**FIGURE 3:**
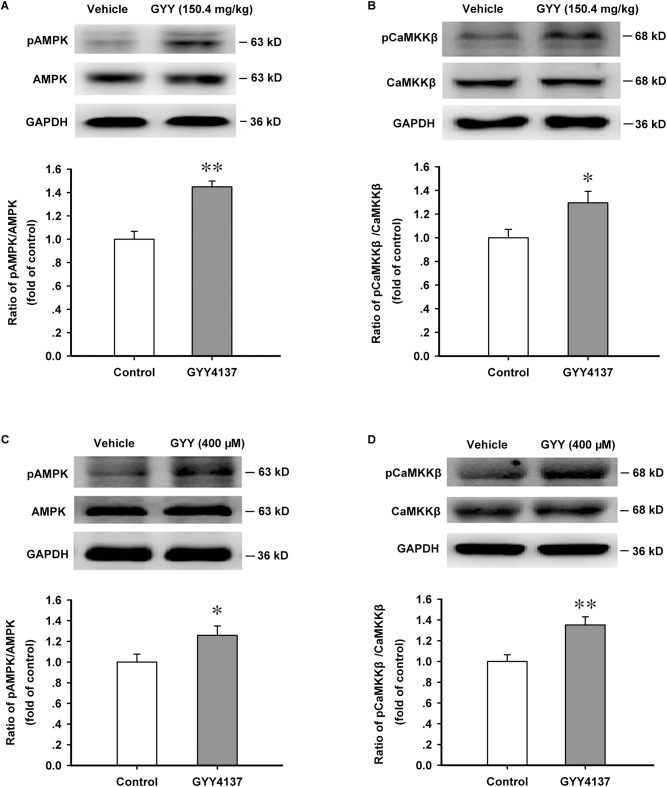
GYY4137 enhances the activation of AMPK and CaMKKβ in ARC tissue and neurons. **(A)** Western blot assay showing that GYY4137 (150.4 mg/kg) increased the activation level of AMPK in ARC tissue (*n* = 5). **(B)** GYY4137 (150.4 mg/kg) increased the activation level of CaMKKβ in ARC tissue (*n* = 6). **(C)** GYY4137 (400 μM) increased the activation of AMPK in primary culture of ARC neurons (*n* = 5). **(D)** GYY4137 (400 μM) increased the activation of CaMKKβ in primary culture of ARC neurons (*n* = 5). Data are normalized as folds to control and expressed as means ± SEM. One-way ANOVA was used. ^∗^*P* < 0.05, ^∗∗^*P* < 0.01 vs vehicle.

The production of NPY is critically regulated by energy metabolic gage molecule AMPK and CaMKKβ is an important upstream activator of it (Blankenship et al., 2016). By promoting AMPK activation, H_2_S exerts many physiological functions (Chen et al., 2017; Wang et al., 2017). We then explored the effect of GYY4137 on AMPK activation in ARC. We found that GYY4137 (150.4 mg/kg, i.p., 1 h) activated AMPK in ARC region [*n* = 5 mice for each group; vehicle, 1.000 ± 0.068; GYY, 1.449 ± 0.050; *F*_(1,8)_ = 28.474, *P* = 0.001; **Figure 3A**]. GYY4137 (150.4 mg/kg, i.p., 1 h) also activated CaMKKβ (*n* = 6 mice for each group; vehicle, 1.000 ± 0.071; GYY, 1.295 ± 0.097; *F*_(1,10)_ = 5.997, *P* = 0.034; **Figure 3B**]. GYY4137 (400 μM, 1 h) was also administrated in cultured ARC neurons and the western blot results showed that GYY4137 significantly increased the phosphorylation levels of AMPK [*n* = 5 for each group; vehicle, 1.000 ± 0.077; GYY, 1.258 ± 0.090; *F*_(1,8)_ = 5.273, *P* = 0.048; **Figure 3C**] and CaMKKβ [*n* = 5 for each group; vehicle, 1.000 ± 0.065; GYY, 1.351 ± 0.078; *F*_(1,8)_ = 11.975, *P* = 0.009; **Figure 3D**].

### GYY4137 Increases the Protein Sulfur-Sulfhydrylation Level in ARC Tissue

**FIGURE 4:**
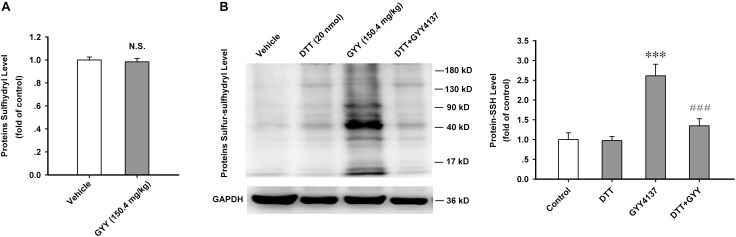
GYY4137 increases the protein sulfur-sulfhydrylation level in ARC tissue. **(A)** GYY4137 (150.4 mg/kg) did not influence the total protein sulfhydryl level in ARC tissue of mice (*n* = 10). N.S., no statistical difference. **(B)** GYY4137 (150.4 mg/kg) increased the protein sulfur-sulfhydryl level in ARC tissue and this effect was blocked by DTT (20 nmol for each mouse; *n* = 5). Protein-SSH, protein sulfur-sulfhydryl. Data are normalized as folds to control and expressed as means ± SEM. One-way ANOVA and *post hoc* tests were used. ^∗∗∗^*P* < 0.001 vs vehicle; ^###^*P* < 0.001 vs GYY.

The most important biochemical property of H_2_S is modifying substrate proteins via sulfur-sulfhydrylation, and DTT is a potent bio-active reductant which breaks disulfide bond in proteins (Mustafa et al., 2011; Paul and Snyder, 2012). Sulfur-sulfhydryl contains a disulfide bond and it can be broken by DTT. We want to know whether GYY affects the overall level of protein sulfhydryls or just directly promotes the formation of S-sulfhydration in substrate proteins. Our present study showed that GYY4137 (150.4 mg/kg, i.p., 1 h) did not influence the sulfhydryl level of ARC proteins [*n* = 10 for each group; vehicle, 1.000 ± 0.025; GYY, 0.984 ± 0.028; *F*_(1,18)_ = 0.182, *P* = 0.674; **Figure 4A**]. But GYY4137 very significantly increased the protein sulfur-sulfhydryl level in ARC, which could be completely eliminated by treatment with DTT [20 nmol for each mouse, i.c.v.; *n* = 5 for each group; *F*_(3,16)_ = 15.198, *P* = 0.001; vehicle, 1.000 ± 0.171; DTT, 0.975 ± 0.099, *P* = 0.930 vs vehicle; GYY, 2.613 ± 0.292, *P* < 0.001 vs vehicle; DTT+GYY, 1.347 ± 0.180, *P* = 0.203 vs DTT, *P* < 0.001 vs GYY; **Figure 4B**]. We also found that food restriction for 12 h significantly increased the S-sulfhydrylation levels of ARC proteins [*n* = 5 for each group; control, 1.000 ± 0.099; fasted, 2.422 ± 0.115; *t* = 9.313, *P* < 0.001; **Supplementary Figure S1**]. These results indicate that S-sulfhydrylation may mediate the effects of GYY4137 on ARC proteins and it may also play important role in the regulation of food intake.

### Protein Sulfur-Sulfhydrylation Mediates GYY4137-Induced Activation of AMPK and CaMKKβ

**FIGURE 5:**
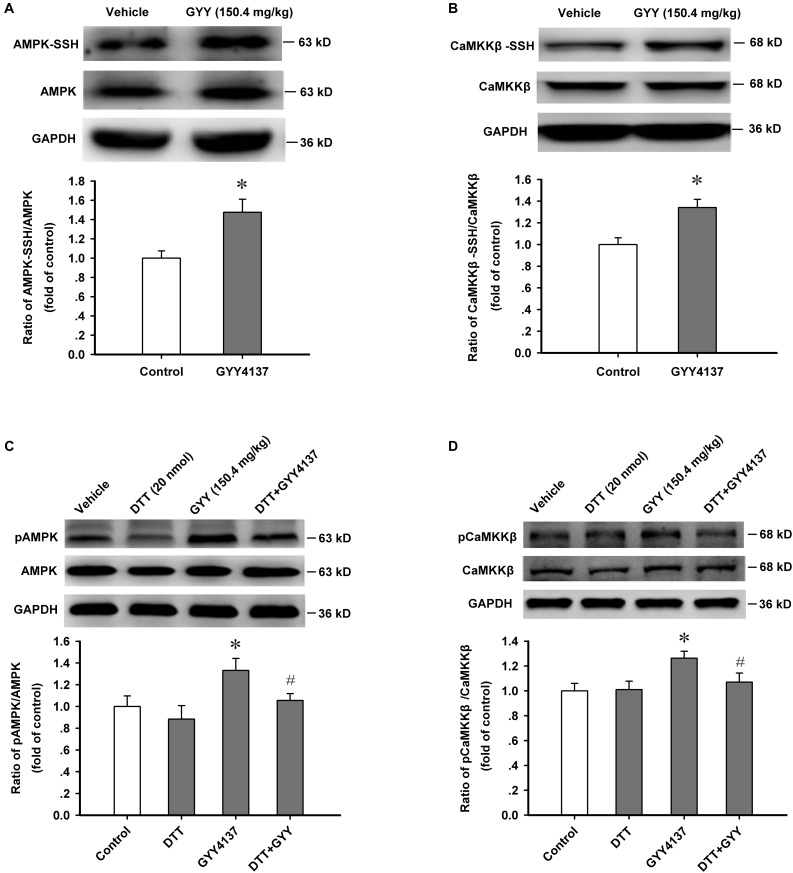
Protein sulfur-sulfhydrylation mediates GYY4137-induced activation of AMPK and CaMKKβ. **(A)** GYY4137 (150.4 mg/kg) increased the sulfur-sulfhydrylation level of AMPK in ARC tissue (*n* = 4). **(B)** GYY4137 (150.4 mg/kg) increased the sulfur-sulfhydrylation level of CaMKKβ in ARC tissue (*n* = 4). **(C)** DTT (20 nmol each mouse) attenuated the increased activation of AMPK induced by GYY4137 (150.4 mg/kg) in ARC tissue (*n* = 4). **(D)** DTT (20 nmol each mouse) attenuated the increased activation of CaMKKβ induced by GYY4137 (150.4 mg/kg) in ARC tissue (*n* = 4). Data are normalized as folds to control and expressed as means ± SEM. One-way ANOVA and *post hoc* tests were used. ^∗^*P* < 0.05 vs vehicle; ^#^*P* < 0.05 vs GYY.

We further detected the sulfur-sulfhydrylation level of AMPK and CaMKKβ, and the effect of protein sulfur-sulfhydrylation on the activation of CaMKKβ/AMPK. Protein sulfur-sulfhydrylation analysis results showed that GYY4137 (150.4 mg/kg, i.p., 1 h) significantly increased the sulfur-sulfhydrylation level of AMPK [*n* = 4 mice for each group; vehicle, 1.000 ± 0.076; GYY, 1.476 ± 0.135; *F*_(1,6)_ = 9.421, *P* = 0.022; **Figure 5A**] and CaMKKβ [*n* = 4 mice for each group; vehicle, 1.000 ± 0.062; GYY, 1.341 ± 0.077; *F*_(1,6)_ = 12.003, *P* = 0.013; **Figure 5B**] in ARC tissue. We also found that treatment with DTT (20 nmol for each mouse, i.c.v.) efficiently attenuated the enhanced activation of AMPK [*n* = 4 mice for each group; *F*_(3,12)_ = 3.834, *P* = 0.038; vehicle, 1.000 ± 0.097; DTT, 0.884 ± 0.125, *P* = 0.357 vs vehicle; GYY, 1.332 ± 0.111, *P* = 0.037 vs vehicle; DTT+GYY, 1.055 ± 0.063, *P* = 0.276 vs DTT, *P* = 0.045 vs GYY; **Figure 5C**] and CaMKKβ [*n* = 4 mice for each group; *F*_(3,12)_ = 4.139, *P* = 0.031; vehicle, 1.000 ± 0.060; DTT, 1.010 ± 0.068, *P* = 0.986 vs vehicle; GYY, 1.263 ± 0.056, *P* = 0.012 vs vehicle; DTT+GYY, 1.071 ± 0.072, *P* = 0.835 vs DTT, *P* = 0.018 vs GYY; **Figure 5D**] induced by GYY4137. In order to confirm the action of H_2_S on AMPK, we added an *in vitro* AMPK activation assay (detected the effect of GYY4137 and DTT on the activation of AMPK in the cultured neurons of ARC, including four groups: Vehicle, DTT 100 μM, GYY 400 μM, DTT+GYY), and found that GYY increased the activity of AMPK, DTT alone did not affect AMPK activation but inhibited the increased AMPK activity induced by GYY (400 μM) *in vitro* [*n* = 4 for each group; *F*_(3,12)_ = 45.58, *P* = 0.001; vehicle, 1.000 ± 0.066; DTT, 0.990 ± 0.043, *P* = 0.896 vs vehicle; GYY, 1.973 ± 0.048, *P* = 0.001 vs vehicle; DTT+GYY, 1.154 ± 0.042, *P* = 0.056 vs DTT, *P* = 0.001 vs GYY; **Supplementary Figure S2**]. This result suggests that protein S-sulfhydrylation plays pivotal roles in GYY-induced AMPK activation. From these results, we could come to the conclusion that protein sulfur-sulfhydrylation has substantive contribution to GYY4137/H_2_S-induced activation of AMPK and CaMKKβ in ARC.

### Protein Sulfur-Sulfhydrylation and AMPK Activation Mediate the Orexigenic Effect of GYY4137

**FIGURE 6:**
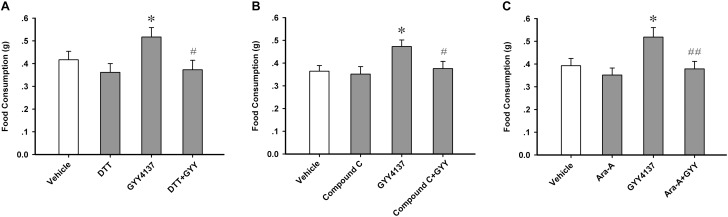
Sulfur-sulfhydrylation and AMPK activation mediate the orexigenic effect of GYY4137. **(A)** DTT (20 nmol each mouse) attenuated the orexigenic effect of GYY4137 (150.4 mg/kg) (*n* = 7). **(B)** AMPK inhibitor Compound C (100 nmol each mouse) prevented the orexigenic effect of GYY4137 (150.4 mg/kg; *n* = 7). **(C)** AMPK inhibitor Ara-A (200 nmol each mouse) prevented the orexigenic effect of GYY4137 (150.4 mg/kg; *n* = 7). Data are expressed as means ± SEM. One-way ANOVA and *post hoc* tests were used. ^∗^*P* < 0.05 vs vehicle; ^#^*P* < 0.05, ^##^*P* < 0.01 vs GYY.

As AMPK activity in the ARC region is critically associated with feeding behavioral outcome (Oh et al., 2016), we investigated whether GYY4137-induced feeding enhancement was mediated by sulfur-sulfhydrylation and the activation of AMPK. Sulfur-sulfhydrylation blocker DTT (20 nmol each mouse, i.c.v.) and AMPK inhibitors, Compound C (100 nmol each mouse, i.c.v.) and Ara-A (200 nmol each mouse, i.c.v.), were administrated 10 min before GYY4137 (150.4 mg/kg, i.p.). Then the food consumption of each mouse was tested during the 2 h after GYY4137 application. The feeding study results showed that DTT [*n* = 7 mice for each group; *F*_(3,24)_ = 3.177, *P* = 0.042; vehicle, 0.417 ± 0.037; DTT, 0.361 ± 0.039, *P* = 0.332 vs vehicle; GYY, 0.517 ± 0.041, *P* = 0.044 vs vehicle; DTT+GYY, 0.373 ± 0.042, *P* = 0.841 vs DTT, *P* = 0.017 vs GYY; **Figure 6A**], Compound C [*n* = 7 mice for each group; *F*_(3,24)_ = 3.440, *P* = 0.033; vehicle, 0.364 ± 0.025; Compound C, 0.351 ± 0.033, *P* = 0.764 vs vehicle; GYY, 0.473 ± 0.029, *P* = 0.017 vs vehicle; Compound C+GYY, 0.376 ± 0.032, *P* = 0.571 vs Compound C, *P* = 0.031 vs GYY; **Figure 6B**] or Ara-A [*n* = 7 mice for each group; *F*_(3,24)_ = 4.547, *P* = 0.012; vehicle, 0.393 ± 0.032; Ara-A, 0.352 ± 0.031, *P* = 0.408 vs vehicle; GYY, 0.518 ± 0.042, *P* = 0.016 vs vehicle; Ara-A+GYY, 0.379 ± 0.033, *P* = 0.586 vs Ara-A, *P* = 0.009 vs GYY; **Figure 6C**] alone did not influence food intake, but they significantly attenuated the promoting effect of GYY4137 on feeding behavior of mice. To exclude the peripheral effects of systemic administration of GYY, we carried out experiments in which GYY was administered via i.c.v. infusion (100 nmol each mouse, **Supplementary Figure S3A**) to confirm its behavioral and molecular effects, including ARC protein S-sulfhydrylation, AMPK activation, and feeding. We found that GYY via i.c.v. infusion also significantly increased the levels of ARC protein S-sulfhydrylation (*n* = 5 mice per group; vehicle, 1.000 ± 0.069; GYY, 2.788 ± 0.172; *t* = 9.638, *P* < 0.001; **Supplementary Figure S3B**), AMPK activation (*n* = 5 mice per group; vehicle, 1.000 ± 0.034; GYY, 1.437 ± 0.040; *t* = 8.379, *P* = 0.001; **Supplementary Figure S3C**) in 1 h and food intake in 2 h (*n* = 8 mice per group; vehicle, 0.370 ± 0.039; GYY, 0.526 ± 0.044; *t* = 2.651, *P* = 0.019; **Supplementary Figure S3D**). Taken together, these results suggest that GYY4137/H_2_S stimulates feeding behavior via sulfur-sulfhydrylation and sequently the enhancement of AMPK activation in ARC.

## Discussion

In the present study, we found that GYY4137-derived H_2_S has orexigenic effect on mice and the possible underlying mechanisms. Our investigation showed that GYY4137 could promote mice food intake, augment the production of NPY and simultaneously, enhance the sulfur-sulfhydrylation and activation of AMPK and CaMKKβ in ARC. Blocking sulfur-sulfhydrylation with DTT eliminated GYY4137-induced activation of AMPK and CaMKKβ. DTT and preventing AMPK activation in ARC with Compound C amd Ara-A, could both attenuate the orexigenic effect of GYY4137.

We used GYY4137 as a H_2_S donor to investigate the possible effect of H_2_S on ARC and food intake of mice. We found that GYY4137/H_2_S increased food consumption within the physiological concentration range of H_2_S (50–200 μM, with GYY4137 at 100–800 μM). GYY4137 is a water soluble, slow, and stable releasing H_2_S donor. For example, with a single dose of GYY4137 at 100 μM, the concentration of GYY4137-derived H_2_S can keep at 20 μM for about 1–5 h *in vivo* (Rose et al., 2015). The optimal observation time for GYY action is within 1–5 h after treatment. Since repeated administration via i.p. injection may induce adverse stimulation to mice, we used a single dose treatment and tested the food consumption of mice during the 1–8 h period. In our study, the orexigenic effect of a single dose of GYY4137 maintained for 4 h, and at 8 h after GYY4137 administration the orexigenic effect disappeared. Thus, the time-dependence of the orexigenic effect of GYY4137 is probably associated with its chemical and H_2_S-releasing property. Besides, the highest dose of GYY in our study is 300.8 mg/kg, which is a relatively safe dose lower than the toxic amount but could also induce some unexpected neuroexcitable effects such as increased locomotive activity, epilepsy, hypertension, and pain sensitization (Feng et al., 2013; Kuksis et al., 2014; Luo et al., 2014), and that may affect the feeding behavior of the mice under this treatment level.

Modification of substrate proteins by sulfur-sulfhydrylation is a principal action mode of H_2_S (Mustafa et al., 2011; Paul and Snyder, 2012). We hypothesized that sulfur-sulfhydrylation of certain target proteins in ARC is the foundation of the orexigenic effect of H_2_S. DTT is a blocker of S-sulfhydrylation, which is commonly used in blocking the S-sulfhydrylation and functions of H_2_S (Mustafa et al., 2011; Paul and Snyder, 2012; Sen, 2017). Our results showed that GYY4137/H_2_S significantly increased the proteins sulfur-sulfhydryl level, which could be blocked by DTT. This result confirmed that DTT could be used as an efficient sulfur-sulfhydrylation blocker. AMPK in ARC plays critical roles in the control of appetite and feeding behavior (Oh et al., 2016; Dong et al., 2017). Our experiments found that DTT attenuated the activation of AMPK and CaMKKβ induced by GYY4137, and DTT and AMPK inhibitors both successfully attenuated the orexigenic effect of GYY4137. So we deemed protein sulfur-sulfhydrylation substantially contributory to H_2_S-induced CaMKKβ/AMPK activation and food intake. The sulfur-sulfhydrylation effect of H_2_S can be quickly abolished by the breakage of sulfur-sulfhydryl bond, which is a brittle and reversible chemical bond (Mustafa et al., 2011; Paul and Snyder, 2012). We found that the enhanced sulfur-sulfhydrylation in ARC caused by GYY4137 was almost completely blocked by the administration of DTT, which meat DTT given through i.c.v. injection is efficient in controlling sulfur-sulfhydrylation of ARC proteins. Feeding study also showed that DTT significantly attenuated the orexigenic effect of GYY4137 on mice, and thus demonstrated that GYY4137-derived H_2_S stimulates feeding behavior via signaling regulation involving protein sulfur-sulfhydrylation.

AMPK is a critical gage molecule in the monitoring of energy status and controlling of energy metabolism (Kohno et al., 2011; Oh et al., 2016; Dong et al., 2017). In the ARC region, AMPK mainly determines the activity of glucose sensitive neurons and regulates the secretion of orexigenic neuropeptide such as NPY, which is the most abundant neuropeptide in brain and an important appetite-promoting hormone (Kohno et al., 2011; Blankenship et al., 2016; Carneiro et al., 2016). Calcium signal also plays pivotal roles in the production of NPY and energy metabolism (Anderson et al., 2008; Wu et al., 2013). Accumulated evidence suggests that H_2_S is an important functional regulator of several ion channels (Peers et al., 2012; Zhang and Bian, 2014) and influences the intracellular calcium signals (Lee et al., 2006; Tang et al., 2008; Marino et al., 2016). Ca^2+^ is an important activator of CaMKKβ/AMPK signaling cascade (Wu et al., 2013; Nakanishi et al., 2017), and H_2_S activates CaMKKβ/AMPK signaling in many kinds of cells (Lee et al., 2012; Zhou et al., 2014). Therefore, we suppose that H_2_S augments NPY content and food intake via increasing intracellular calcium level and activating CaMKKβ/AMPK signaling in ARC orexigenic neurons, though the enhancement of calcium signals in orexigenic neurons needs to be proved in further study.

From this study, we can also draw out some other questions for the future investigations. First, is inhaling a proper dose of H_2_S gas capable of inducing the orexigenic effect? Second, whether endogenous H_2_S and its sulfur-sulfhydrylation function are involved in the regulation of food intake? It has been reported that fasting or diet can increase the endogenous production of H_2_S (Hine and Mitchell, 2015; Hine et al., 2015; Nakano et al., 2015). So we can suppose that endogenous H_2_S, to some extent, may be implicated in refeeding increase induced by energy stress such as starvation or fasting, and perhaps protein sulfur-sulfhydrylation is involved in the process of the orexigenic effect. Third, since H_2_S influences intracellular Ca^2+^ level in many cells and calcium signal is a critical activating factor for hypothalamic CaMKKβ/AMPK (Lee et al., 2012; Wu et al., 2013; Zhou et al., 2014; Chen et al., 2017), which is involved in the regulation of energy balance (Anderson et al., 2008), is calcium signal also contributory for the enhanced activation of CaMKKβ/AMPK in ARC and the increased food intake caused by exogenous H_2_S? Therefore, whether H_2_S may activate AMPK through augmenting intracellular calcium signal in ARC, and whether sulfur-sulfydrylation has substantial contribution to this process still need further research. What is more, unbiased identification of the abundant S-sulfhydrated proteins in the GYY4137-treated arcuate lysate by bidirectional electrophoresis and bio-mass spectrometry would represent an important future direction and will help in finding special sites for S-sulfhydrylation in individual and specific proteins.

## Conclusion

In summary, we confirmed the orexigenic effect of H_2_S by means of GYY4137 administration and its possible mechanism via sulfur-sulfhydrylation of certain functional proteins. GYY4137-derived H_2_S increases the activation of energy metabolic gage AMPK and its upstream kinase CaMKKβ via sulfur-sulfhydrylation, further enhances the activity of the orexigenic neurons in ARC including NPY neurons, and subsequently promotes appetite. This study provides foundation for the investigation of the roles of endogenous H_2_S in the hypothalamus and energy metabolism which influences food intake, and the function of protein sulfur-sulfhydrylation on the production of appetite-regulating neuropeptides including NPY. Besides, our investigation also suggests that H_2_S-derived modulating agents may be of value in the design and development of therapeutic medications for diseases such as anorexia nervosa.

## Author Contributions

JZ designed and substantially performed the experiments, analyzed the data, and wrote the paper. X-HL, BG, and L-YD helped in biochemical detections and provided expert advice. KD, J-JF, and A-QS helped in animal feeding and experiments. W-NW designed and oversaw the experiments, analyzed and interpreted the data, and co-wrote and developed the manuscript.

## Conflict of Interest Statement

The authors declare that the research was conducted in the absence of any commercial or financial relationships that could be construed as a potential conflict of interest. The reviewers JB and DM and handling Editor declared their shared affiliation.
