# Supplementary material for: GYY4137 Promotes Mice Feeding Behavior via Arcuate Nucleus Sulfur-Sulfhydrylation and AMPK Activation
Source: Front Pharmacol. 2018 Aug 21;9:966. doi: 10.3389/fphar.2018.00966 (PMC6111581; doi:10.3389/fphar.2018.00966)
Supplement: Supplementary file 1 [file Data_Sheet_1.PDF]

GYY4137 promotes mice feeding behavior via arcuate nucleus S-sulfhydrylation and AMPK activation

Jun Zhou, Xiao-Hui Lv, Jun-Juan Fan, Li-Yun Dang, Kun Dong, Bo Gao, Ao-Qi Song, Wen-Ning Wu

Supplementary Material

Supplementary Figures (Figure S1-S3) and Figure legends

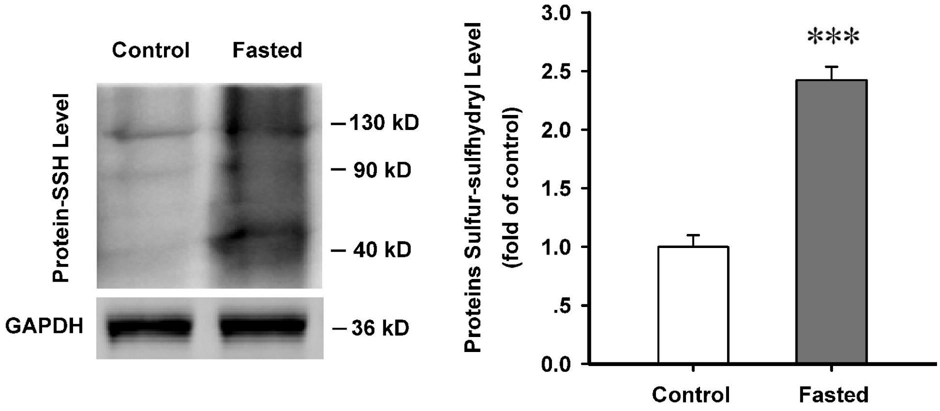

Figure S1 Food restriction increases the ARC protein S-sulfhydrylation level in mice

Food restriction for 12 h significantly increased the S-sulfhydrylation levels of ARC proteins (n = 5 for each group). Data are normalized as folds to control and expressed as means ± SEM. Independent and two-side t-test was used. *** $P < 0.001$ vs control. This figure is a 1-column fitting image.

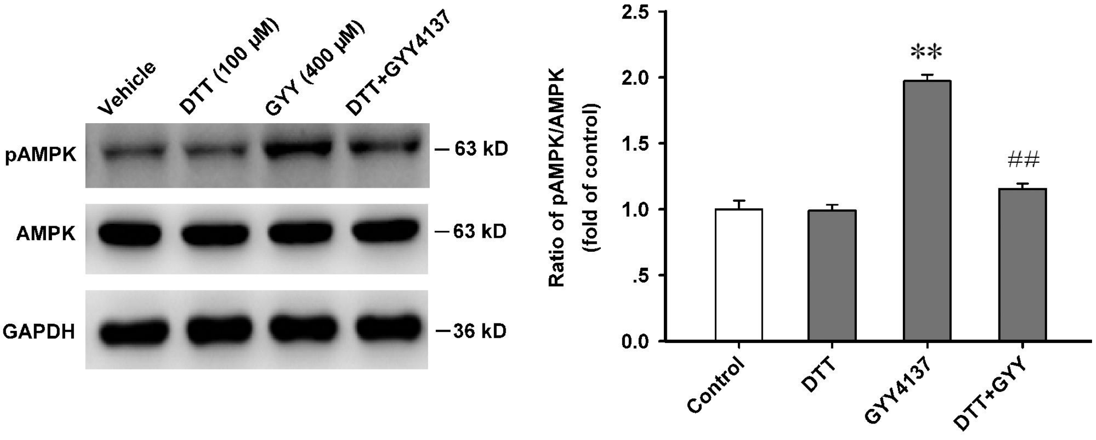

Figure S2 GYY4137 activates AMPK on cultured ARC neurons in a S-sulfhydrylation-dependent manner

GYY (400 μM) significantly increased the activity of AMPK. DTT (100 μM) alone did not affect the activation of AMPK but it effectively inhibited the increased AMPK activity induced by GYY in vitro (n = 4 for each group). Data are normalized as folds to control and expressed as means ± SEM. One-way ANOVA and post hoc tests were used. ** $P < 0.01$ vs vehicle; ## $P < 0.01$ vs GYY. This figure is a 1-column fitting image.

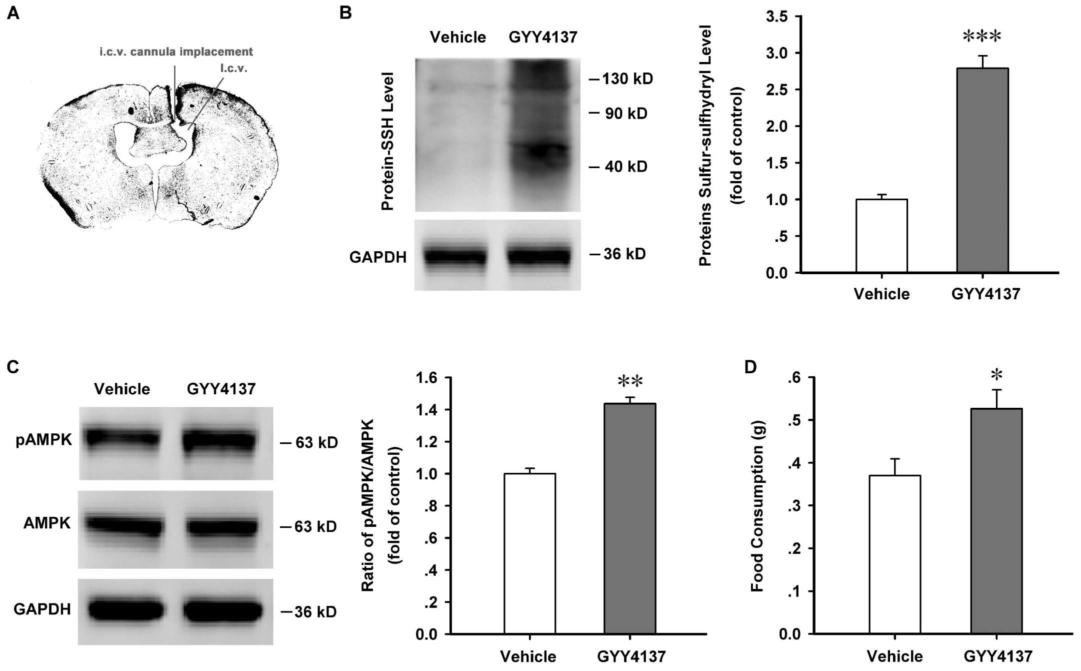

Figure S3 The effects of GYY4137 administrated via lateral cerebral ventricle

infusion on mice

(A) A brain slice photo of mouse after i.c.v. cannula implacement, fixation and slicing, which shows the lateral cerebral ventricle and the trace of i.c.v. cannula implanting. (B-D) GYY administrated through i.c.v. infusion (100 nmol each mouse) significantly enhanced the level of ARC protein S-sulfhydrylation (n = 5 mice for each group), the level of AMPK activation (n = 5 mice for each group) in 1 h, and food intake in 2 h (n = 8 mice for each group). Data are normalized as folds to control and/or expressed as means ± SEM. Independent and two-side t-test was used. * $P < 0.05$, ** $P < 0.01$, *** $P < 0.001$ vs control. This figure is a 2-column fitting image.
